# Dysregulation of Serum MicroRNA after Intracerebral Hemorrhage in Aged Mice

**DOI:** 10.3390/biomedicines11030822

**Published:** 2023-03-08

**Authors:** Dominic Robles, De-Huang Guo, Noah Watson, Diana Asante, Sangeetha Sukumari-Ramesh

**Affiliations:** Department of Pharmacology and Toxicology, Medical College of Georgia, Augusta University, 1120 15th Street, CB3618, 30912 Augusta, Georgia; dominic.s.robles@live.mercer.edu (D.R.); dehuangguo@yahoo.com (D.-H.G.); nowatson@augusta.edu (N.W.); dasante@augusta.edu (D.A.)

**Keywords:** intracerebral hemorrhage, aging, microRNA

## Abstract

Stroke is one of the most common diseases that leads to brain injury and mortality in patients, and intracerebral hemorrhage (ICH) is the most devastating subtype of stroke. Though the prevalence of ICH increases with aging, the effect of aging on the pathophysiology of ICH remains largely understudied. Moreover, there is no effective treatment for ICH. Recent studies have demonstrated the potential of circulating microRNAs as non-invasive diagnostic and prognostic biomarkers in various pathological conditions. While many studies have identified microRNAs that play roles in the pathophysiology of brain injury, few demonstrated their functions and roles after ICH. Given this significant knowledge gap, the present study aims to identify microRNAs that could serve as potential biomarkers of ICH in the elderly. To this end, sham or ICH was induced in aged C57BL/6 mice (18–24 months), and 24 h post-ICH, serum microRNAs were isolated, and expressions were analyzed. We identified 28 significantly dysregulated microRNAs between ICH and sham groups, suggesting their potential to serve as blood biomarkers of acute ICH. Among those microRNAs, based on the current literature, miR-124-3p, miR-137-5p, miR-138-5p, miR-219a-2-3p, miR-135a-5p, miR-541-5p, and miR-770-3p may serve as the most promising blood biomarker candidates of ICH, warranting further investigation.

## 1. Introduction 

Stroke is one of the most severe health issues that plagues the healthcare system. Intracerebral hemorrhage (ICH) is the second most common type of stroke and has a higher risk of mortality and morbidity rates than other stroke types [1]. Notably, there is no effective treatment for ICH [2,3,4,5]. Therefore, preclinical and clinical research on this disease is essential. ICH arises in the form of blood vessel rupture in the brain, resulting in the accumulation of blood in the brain parenchyma and the development of hematoma [6]. ICH often causes severe brain damage that is categorized into primary and secondary brain injuries. The mass effect of the hematoma mostly contributes to primary brain damage, whereas the oxidative and inflammatory signaling pathways [7,8], induced by blood components such as thrombin, hemoglobin, hemin, and iron, are responsible for secondary brain damage [9,10]. In contrast to primary brain damage, secondary brain damage persists for a longer period of time, which could contribute to both acute and long-term neurological outcomes [11]. Hence, the molecular regulators of secondary brain damage are considered potential targets for therapeutic intervention [12]. However, a detailed mechanistic understanding of the molecular events underlying secondary brain injury after ICH is lacking [13]. This represents a significant gap in the literature and reflects on the lack of defined therapeutic targets.

MicroRNAs (miRNAs), short non-coding RNAs, comprise a group of regulatory molecules that modulate the expression of genes, which play critical roles in cellular processes such as inflammation and apoptosis [14,15]. Many studies have identified the changes in miRNA expression in ischemic stroke [16], while there remains a significant gap in our knowledge of their dysregulation in ICH, particularly in the elderly. Notably, circulating miRNAs undergo dysregulation in response to pathological conditions [17] and can be found in a remarkably stable form in serum or plasma [18]. Therefore, miRNAs could serve as non-invasive diagnostic and prognostic blood biomarkers. Specifically, diagnostic blood biomarkers may help distinguish ICH from ischemic stroke, while prognostic blood biomarkers may be able to predict mortality or poor outcomes after ICH.

Aging is characterized by the accumulation of degenerative processes. MiRNAs contribute to aging [19] and have regulatory roles in neurodegeneration [20,21]. Moreover, aging is listed as the most profound risk factor for cardiovascular and neurological diseases [22]. Notably, ICH incidence and mortality rates increase with aging [23,24,25], but the precise role of aging in the pathophysiology of ICH remains largely unknown. Therefore, it is highly required to characterize the molecular level changes that occur after ICH in aged subjects, as it may help develop novel strategies for the diagnosis and management of ICH. Though preclinical animal models of ICH are invaluable tools for studying disease pathophysiology, miRNA dysregulation post-ICH was mostly studied in young animal subjects [17]. Moreover, aging is associated with miRNA expression level changes in mice and humans [26,27,28,29]. Hence, the objective of this study is to identify circulating miRNAs that are dysregulated after ICH in aged mice, as it may help characterize the pathophysiology of ICH in the elderly.

## 2. Methods

### 2.1. ICH Induction

All animal studies were performed according to the protocols approved by the Institutional Animal Care and Use Committee, in accordance with the NIH and USDA guidelines. Intracerebral hemorrhage was induced in aged male C57BL/6 mice (18–24 months), (Jackson Laboratories, Bar Harbor, ME, USA), as previously reported by our laboratory [2,30,31,32,33]. Briefly, mice were anesthetized with isoflurane and positioned prone on a stereotaxic head frame (Stoelting, Wood Dale, IL, USA). Using a high-speed drill (Dremel, Racine, WI, USA), a burr hole (0.5 mm) was made 2.2 mm lateral to the bregma, and a small animal temperature controller (David Kopf Instruments, Los Angeles, CA, USA) was used to keep the body temperature at 37 ± 0.5 °C. Employing a Hamilton syringe (26-G), 0.04 U of bacterial type IV collagenase (Sigma, St. Louis, MO, USA) in 0.5 μL phosphate-buffered saline (phosphate buffered saline; pH 7.4 (PBS) was injected with the stereotaxic guidance 3.0 mm into the left striatum to induce ICH [2]. After removing the needle, bone wax was used to seal the burr hole and the incision was stapled. Sham mice underwent the same surgical procedure, but only PBS (0.5 μL) was injected, which served as the experimental control.

### 2.2. Neurobehavioral Analysis

Mice were analyzed for neurobehavioral deficits, as previously reported, using a 24-point scale [33,34,35], which estimates sensorimotor deficits. The neurobehavioral analysis consisted of six different tests: circling, climbing, beam walking, compulsory circling, bilateral grasp, and whisker response. Each test was graded from 0 (no impairment) to 4 (severe impairment) and the sum of the scores on all six tests established a composite neurological deficit score.

### 2.3. Serum Collection

Blood was collected from deeply anesthetized mice and allowed to clot, undisturbed, at room temperature. Then, the clot was removed by centrifugation at 1500× *g* for 10 min in a refrigerated centrifuge. The supernatant or serum was collected and stored at −80 °C. Before miRNA isolation, the serum was thawed and centrifuged, and the supernatant was used for miRNA isolation.

### 2.4. miRNA Isolation

miRNA isolation procedure was performed using the miRNeasy Mini Kit (Qiagen, Hilden, Germany, catalogue. No: 217004), according to the manufacturer’s instructions, with some modifications. Briefly, the TRIzol LS reagent was added to mouse serum (0.75 mL TRIzol per 0.25 mL serum). This was followed by the addition of chloroform (0.2 mL chloroform per 0.75 mL of TRIzol), and centrifugation at 12,000× *g* at 4 °C for phase separation. The aqueous phase was transferred to a new tube and 100% ethanol (1.5 volumes of the sample) was added and mixed thoroughly and transferred to the RNeasy Mini spin column to elute the miRNA, according to the manufacturer’s instructions.

### 2.5. miRNA Sequencing

RNA quality and quantity were assessed by the Agilent 2100 bioanalyzer (Agilent Technologies, Santa Clara, CA, USA). Purified small RNA samples were processed for cDNA library preparations using the QIAseq miRNA Library kit (Qiagen, catalogue. No: 331502). Briefly, 15 ng of purified small RNA was ligated with a 3′ adaptor and 5′ adaptor, and converted to cDNA using RT primer with integrated unique molecular indices (UMI), to enable the quantification of individual miRNA molecules. The cDNA products were purified, enriched with PCR, and purified using QMN Beads (Qiagen, catalog. No: 331502) to create the final cDNA library. The prepared library was examined by a bioanalyzer and Qubit (Thermo Fisher, Waltham, MA, USA), to test the quality and quantity of the sequencing library, respectively. The libraries were pooled with the correspondingly identified bar codes for each sample and run on the NextSeq500 sequencing system using a 75-cycle paired-end protocol. BCL files generated by the NextSeq500 were converted to FASTQ files for downstream analysis. Reads that passed quality control with individual UMI counts were aligned to the murine reference miRNA sequences using a web-based tool, GeneGlobe Data Analysis Center of QIAGEN, which also performed differential expression analysis and generated a volcano plot, and a hierarchical clustering heatmap.

### 2.6. Statistical Analysis

Statistical analysis was performed using GraphPad Prism software and the student’s *t*-test was used for two-group comparisons. *p* < 0.05 was taken as statistically significant.

## 3. Results

### 3.1. Serum microRNA Isolation and Analysis after ICH

**Figure 1:**
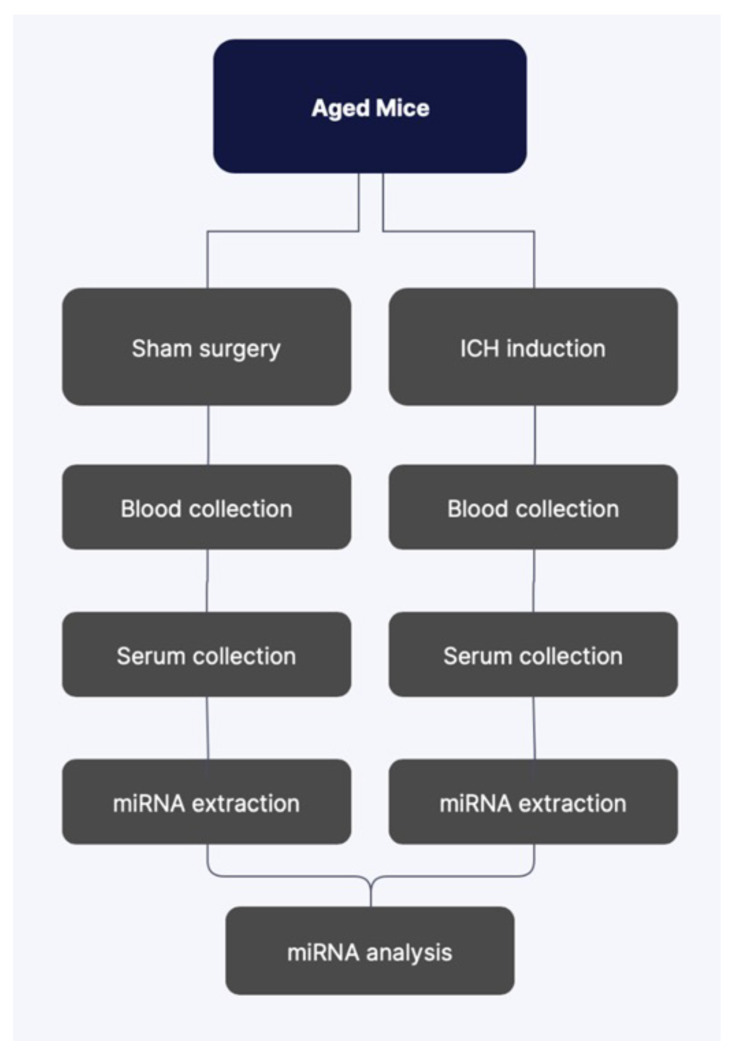
Schematic representation of the overall experimental design. Sham or ICH was induced in aged, male mice. Blood was collected at 24 h post-sham/ICH and serum miRNAs were extracted and subjected to microRNA sequencing.

**Figure 2:**
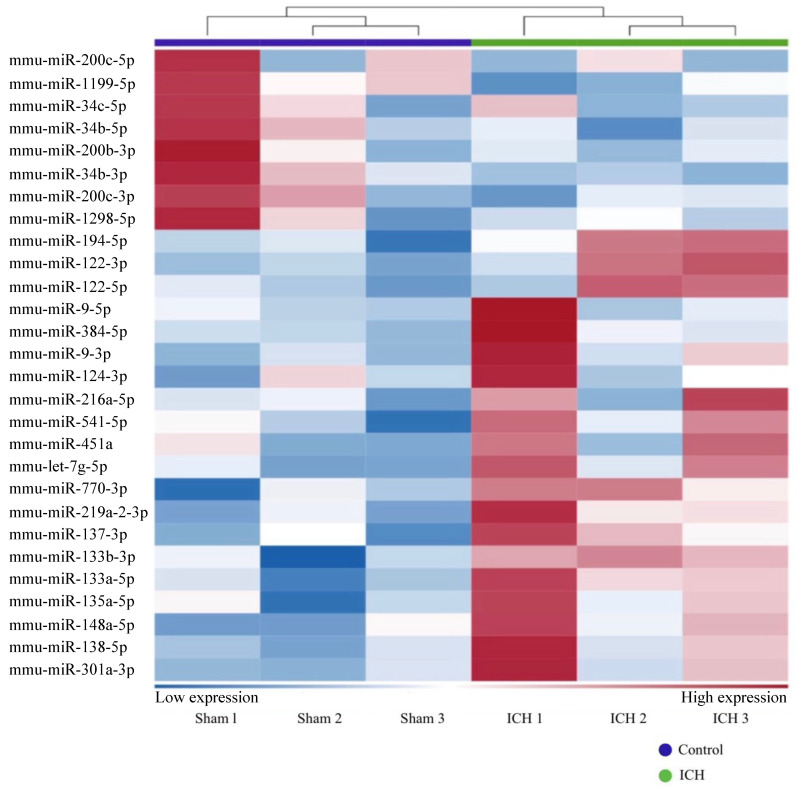
Heatmap representation of the differentially expressed serum miRNAs between sham and ICH. A total of 28 miRNAs exhibited a difference (*p* < 0.05) in their expression between ICH and sham. The serum levels of 20 miRNAs were found to be significantly increased and serum levels of 8 miRNAs were significantly decreased after ICH in comparison to sham (n = 3 mice per group; *p* < 0.05).

**Figure 3:**
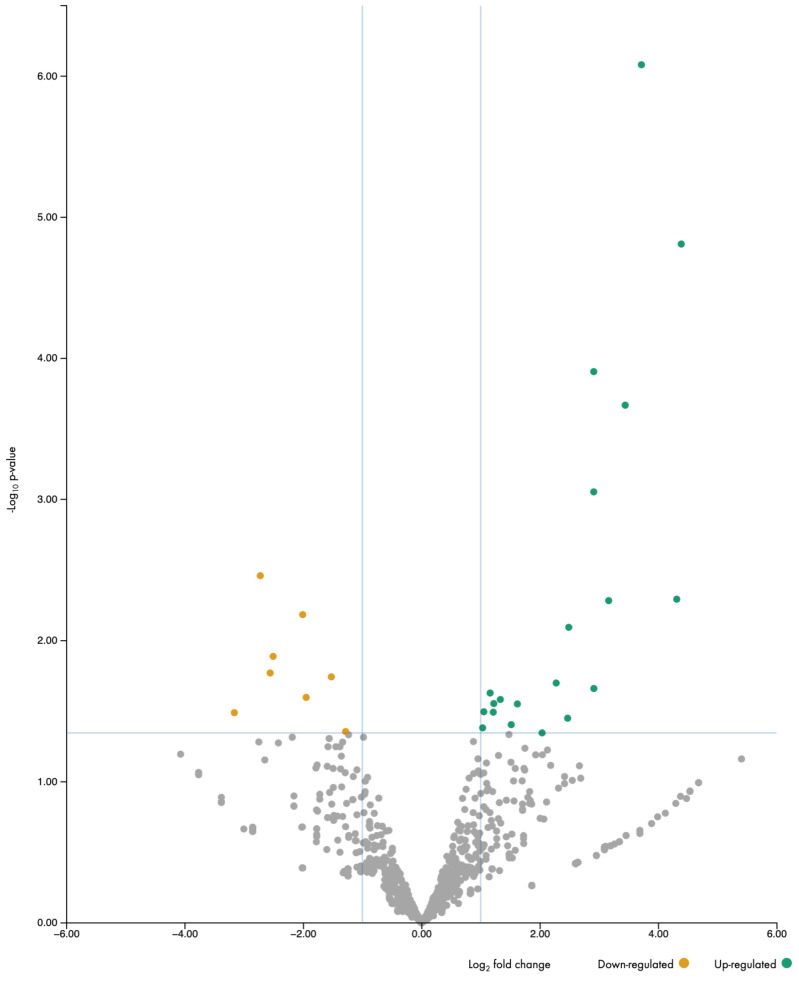
A volcano plot demonstrating dysregulated miRNAs after ICH, compared to sham. A total of 28 miRNAs exhibited a difference (*p* < 0.05) in their expression between ICH and sham. The serum levels of 20 miRNAs were found to be significantly increased (green dots) and serum levels of 8 miRNAs were significantly decreased (yellow dots) after ICH, in comparison to sham (*p* < 0.05; fold-change ≥ 2).

**Figure 4:**
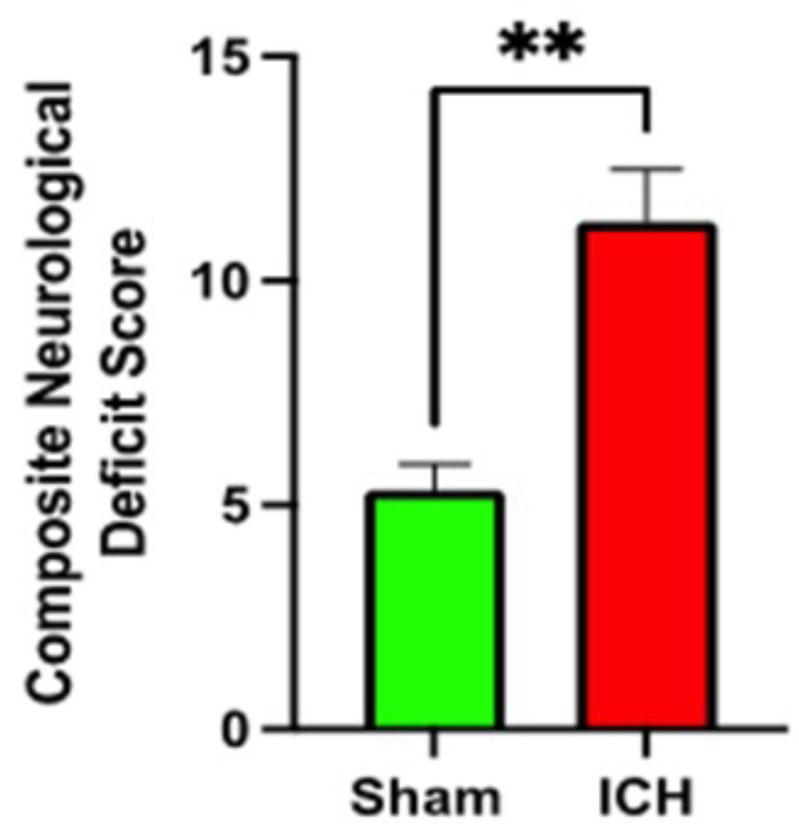
Induction of ICH in aged, male C57BL/6 (18–24 months) mice resulted in significant neurological deficits in comparison to sham. This was estimated using a 24-point scale, as described in methods, on day 1 post-surgery (*n* = 3 animals per group ** *p* < 0.01 vs. sham).

ICH was induced in the striatum of aged, male C57BL/6 (18–24 months) mice, using the collagenase injection method. For miRNA analysis, we collected whole blood from mice on day 1 post-ICH, an acute time point, which exhibited profound neurodegeneration [36] and had significant predictive values in the patient prognosis [37]. Serum microRNA was then isolated, as described in the methods, and subjected to RNA sequencing using the Agilent 2100 bioanalyzer (Agilent Technologies). The serum miRNAs from sham animals served as the experimental controls and the schematic representation of the overall experimental design is depicted (Figure 1). The analysis of RNA sequencing data, using QIAGEN GeneGlobe Data Analysis Center, identified 1960 miRNAs, out of which 28 miRNAs exhibited a significant difference (*p* < 0.05) in their expression between ICH and sham (Table 1, Figure 2 and Figure 3). Among those, the serum levels of 20 miRNAs were found to be significantly increased (*p* < 0.05) and the serum levels of 8 miRNAs were significantly decreased (*p* < 0.05) after ICH in comparison to sham (Table 1). Soon before collecting the blood samples for miRNA analysis, the animals were subjected to neurobehavioral analysis to confirm the ICH induction. Notably, ICH animals exhibited profound neurobehavioral deficits in comparison to sham (*p* < 0.01; Figure 4).

### 3.2. Functional Annotation of Differentially Expressed microRNAs

Many of the dysregulated miRNAs identified in this study play roles in various pathological conditions, as shown in Table 2. Notably, miR-122-5p, miR-9-3p, miR-9-5p, miR-137-3p, miR-1298-5p, miR-219a-2-3p, miR-384-5p, miR-124-3p, miR-34b-5p, miR-200b-3p, miR-135a-5p, miR-133b-3p, miR-1199-5p, miR-451a, miR-138-5p, miR-146a-5p, miR-200b-5p, and miR-483-5p have roles in neuroinflammation, oxidative stress, and apoptosis, which are critical processes associated with secondary brain damage after ICH.

### 3.3. The Comparative Analysis of miRNAs

**Figure 5:**
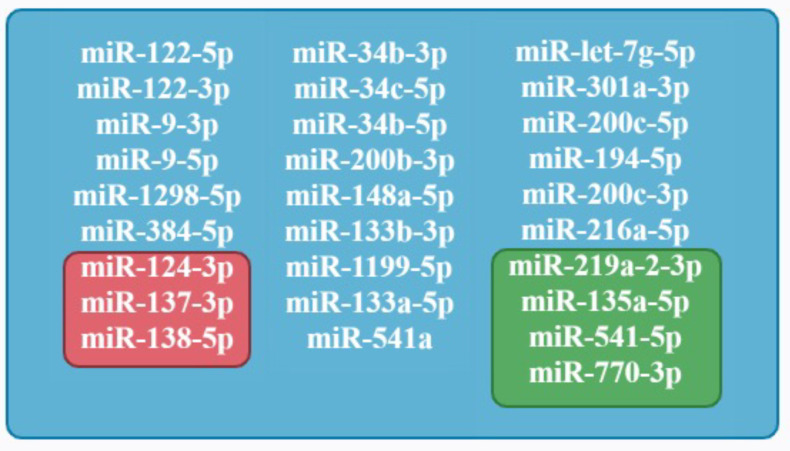
Comparative analysis of dysregulated serum miRNAs after ICH in aged mice (blue square) with human-brain-enriched miRNAs (red square) and dysregulated plasma miRNAs after ICH in young rats (green square). The serum microRNAs, which are human-brain-specific and differentially regulated in the plasma of young mice after ICH, are indicated.

To determine the clinical significance of our observations, we compared our results to the study reported by Wang et al. [75], which enabled the comparison of dysregulated serum miRNAs after ICH in aged mice with human-brain-enriched miRNAs and plasma miRNAs after ICH in young rodents, as demonstrated in Figure 5. We found that miR-124-3p, miR-138-5p, and miR-137-5p, which are differentially expressed in the aged mouse serum after ICH, are also enriched in the human brain tissue, implicating their potential role in ICH-associated brain injury or recovery. Also, the miR-135a-5p level was increased in the blood of both aged mice and young rats after ICH, while miR-219a-2-3p, miR-770-3p, and miR-541-5p were differentially dysregulated in aged mice and young rats after ICH. This discrepancy could be due to differences in the age of the animal models or species that were used in preclinical studies. Therefore, further research is needed to validate these findings as these microRNAs could serve as invaluable molecular targets for the diagnosis and management of ICH that occurs in the elderly.

## 4. Discussion

We identified 28 significantly dysregulated miRNAs in the serum of aged mice after ICH. In comparison to a previous study by Wang et al., [75], miR-124-3p, miR-137-5p, miR-138-5p, miR-219a-2-3p, miR-135a-5p, miR-541-5p, and miR-770-3p could be the most potential candidates to be tested for their roles post-ICH in the elderly. Wang et al. [75] demonstrated the dysregulation of plasma miRNAs in a young rat model of ICH. They focused primarily on miR-124 as a biomarker for ICH, but we also found increased serum levels of miR-138-5p and miR-137-5p in aged mice after ICH. A recent study documented an increased serum level of miR-137 after traumatic brain injury in patients [45]. Moreover, miR-124-3p, miR-137-5p, and miR-138-5p are human-brain-specific miRNAs [75], further demonstrating their potential to serve as serum biomarkers or therapeutic targets for intracerebral hemorrhage, warranting further investigation. Overall, the functional roles of miR-124-3p, miR-137-5p, miR-138-5p, miR-219a-2-3p, miR-135a-5p, miR-541-5p, and miR-770-3p in various pathological processes/states and their possible functions in the pathophysiology of ICH are discussed.

### 4.1. miR-124-3p

We found a significant increase in the level of miR-124-3p in the serum of aged mice after ICH compared to sham. This observation is consistent with a previous study, where the miR-124 level was found to be significantly increased in the plasma of ICH patients in the acute phases of injury, suggesting that miR-124 may serve as a biomarker for the diagnosis of ICH [75]. Functionally, miR-124 plays a key role in iron metabolism and neuronal cell death after ICH, and its inhibition reduced brain injury after ICH in aged mice [76], implicating the detrimental role of miR-124 after ICH. Consistently, high serum miR-124 levels were correlated with poor neurological scores in aged ICH patients [76]. Since miR-124-3p is one of the most abundant brain-specific microRNAs, studies are required to elucidate whether brain injury leads to its release into the blood plasma or serum after ICH.

In young rats, miR-124 was significantly elevated in the plasma and brain tissue, in a collagenase-injection mouse model of ICH, during the acute phase of the injury [75]. By contrast, in a blood-injection mouse model of ICH in young mice, miR-124 expression was found to be decreased in the perihematomal region of the brain [77]. Moreover, miR-124 attenuated ICH-induced inflammatory brain damage in young mice by modulating microglia polarization, implicating the neuroprotective role of miR-124 after ICH [77]. The underlying cause of this discrepancy in its expression after ICH and its function could be the difference in the ICH model and the age of animal subjects. Hence, further investigation is highly needed.

Altered serum expression of miR-124 is associated with various brain injuries. To this end, miR-124-3p was not detectable in healthy volunteers, but its increased level was observed in the serum of patients with severe traumatic brain injury [70]. Moreover, serum miR-124 is significantly enhanced in patients with acute ischemic stroke, where its expression positively correlated with infarct volume and degree of brain damage, as assessed by the National Institutes of Health Stroke Scale [145]. Overall, apart from considering miR-124-3p as a potential biomarker candidate for ICH, its precise functional role in the pathophysiology of ICH requires further validation.

### 4.2. miR-137-3p

Our findings show the increased level of miR-137-3p in the serum of aged mice after ICH in comparison to sham. Upregulation of miR-137-3p inhibited neuronal death, parthanatos, a type of programmed cell necrosis associated with ICH [52,146,147,148,149]. In addition, upregulation of miR-137-3p resulted in neuroprotective effects by decreasing neuronal nitric oxide synthase-positive cells and the death of motor neurons after avulsion injury to the spinal cord in rats [53]. Furthermore, an increased level of miR-137 is observed in the serum after traumatic brain injury [45]. Given the role of miR-137-3p in neuronal death and oxidative damage, further studies are warranted to explore its potential as a therapeutic target for ICH.

### 4.3. miR-138-5p

As per the current study, miR-138-5p levels were found to be significantly increased in the serum of aged mice after ICH compared to sham. Notably, breast cancer cell-derived miR-138-5p has been shown to inhibit M1 polarization and promote M2 polarization of macrophages [111]. It has also been proposed as a potential blood biomarker of Parkinson’s disease [114]. Furthermore, miR-138-5p downregulated NLRP3 inflammasome and its downstream gene targets in lipopolysaccharide-treated rat microglia [112]. Overall, given the role of miR-138-5p in macrophage polarization and inflammation, further studies are required to elucidate its functional role after ICH.

In line with dysregulated plasma miRNAs after ICH in rats [75], levels of miR-135a-5p were increased in the serum of aged mice after ICH, but miR-219a-2-3p, miR-541-5p, and miR-770-3p were differentially dysregulated in aged mice and young rats after ICH. Based on their potential roles in ICH pathology in association with their altered expression, their functions in various pathological conditions are discussed.

### 4.4. miR-135a-5p

M2 microglia-derived extracellular vesicles contained elevated levels of miR-135a-5p, which reduced neuronal autophagy and ischemic brain injury in mice by inhibiting inflammasome signaling [92], suggesting its role in neuroprotection. In contrast, exercise decreased miR-135 levels in adult neural precursor cells and miR-135a-5p inhibition stimulated neurogenesis in the dentate gyrus of aged mice [150]. As well, miR-135a-5p expression in the hippocampus is increased in temporal lobe epilepsy in children [91]. miR-135a-5p mediated proapoptotic effect by inducing cellular apoptosis and reduced cell survival in temporal-lobe epilepsy [91]. Furthermore, its inhibition protected glial cells against epilepsy-induced apoptosis [151]. The miR-135a-5p expression level was significantly decreased in the serum samples of atherosclerosis patients and a mouse model of atherosclerosis [90]. Moreover, overexpression of miR-135a-5p induced a cell cycle arrest and apoptosis, and inhibited the proliferation and migration of vascular smooth muscle cells [90]. Additionally, miR-135 is a tumor suppressor and has been shown as a diagnostic biomarker of colorectal cancer [93]. Overall, given its conflicting roles, further studies are highly needed to establish its function after ICH.

### 4.5. miR-219a-2-3p

Upregulation of miR-219a-2-3p in tissue samples has been linked to anti-inflammatory responses, possibly by modulating NK-kB singling and promoting neuroprotection after spinal cord injury [60]. Serum-derived miR-219a-2-3p has also been shown to be a potential biomarker for traumatic brain injury in mice [58], as well as a peripheral blood biomarker for lung cancer in patients [59]. Given its potential as a biomarker in traumatic brain injury and its roles in neuroprotection and anti-inflammatory responses after a neural injury, miR-219a-2-3p needs to be explored further for its possible roles after ICH.

### 4.6. miR-541-5p

Upregulation of miR-541-5p has been linked to hepatocellular carcinoma [127]. miR-541 also contributes to the modulation of telomerase activity [152] and tumor suppression in non-small cell lung cancer [153]. Apart from that, its functional role after a brain pathology remains enigmatic, requiring investigation.

### 4.7. miR-770-3p

miR-770-3p is a biomarker for aging, as its expression was found to be increased in the serum of aged mice in comparison to young mice [126]. Apart from that, the role of miR-770-3p remains largely understudied. Therefore, further research is vital to determine its functions after ICH.

Though the study reveals several novel candidate miRNAs, some of the identified candidates could be related to ICH irrespective of age, and some could be related to ICH in the context of aging. Therefore, further studies are warranted to identify the age-dependency of those candidates. Moreover, owing to the complexity of aging, the identified candidates, whether related to ICH in an age-dependent or -independent manner, require characterization in aged animal subjects to elucidate their possible roles in apoptosis, neuroinflammation, oxidative stress and secondary brain damage after ICH.

## 5. Conclusions

Herein, we identified seven candidate serum miRNAs, miR-124-3p, miR-138-5p, miR-137-3p, miR-219a-2-3p, miR-135a-5p, miR541-5p, and miR-770-3p, which may have roles in the pathophysiology of ICH in the elderly, warranting further investigation. Among those, miR-124-3p, miR-138-5p, and miR-137-3p may have the greatest potential, as they are human-brain-specific miRNAs and are also implicated in neuronal apoptosis and neuroinflammation. Given the increasing prevalence of the aging population and age-related diseases, such as stroke, the miRNAs identified in this study may serve as invaluable molecular targets for future investigation post-ICH, a neurological disorder without an effective treatment option.

## Figures and Tables

**Table 1:** Serum microRNAs that exhibited differential expression between sham and ICH (*p* < 0.05).

MicroRNA	Fold-Change	*p*-Value
mmu-miR-122-5p	13.10	0.00000083
mmu-miR-122-3p	20.88	0.0000155
mmu-miR-9-3p	7.49	0.000124
mmu-miR-9-5p	10.83	0.000215
mmu-miR-137-3p	7.49	0.000884
mmu-miR-1298-5p	−6.62	0.00346
mmu-miR-219a-2-3p	19.81	0.00508
mmu-miR-384-5p	8.93	0.00520
mmu-miR-34b-3p	−4.03	0.00653
mmu-miR-124-3p	5.59	0.00803
mmu-miR-34c-5p	−5.7	0.01290
mmu-miR-34b-5p	−5.9	0.01691
mmu-miR-200b-3p	−2.88	0.01801
mmu-miR-135a-5p	4.83	0.01994
mmu-miR-148a-5p	7.5	0.02179
mmu-miR-133b-3p	2.23	0.02347
mmu-miR-1199-5p	−3.87	0.02520
mmu-miR-133a-5p	2.51	0.02609
mmu-miR-451a	2.33	0.02788
mmu-miR-138-5p	3.06	0.02806
mmu-let-7g-5p	2.07	0.03187
mmu-miR-301a-3p	2.31	0.03210
mmu-miR-200c-5p	−8.98	0.03236
mmu-miR-770-3p	5.52	0.03542
mmu-miR-541-5p	2.85	0.03935
mmu-miR-194-5p	2.04	0.04143
mmu-miR-200c-3p	−2.44	0.04401
mmu-miR-216a-5p	4.09	0.04500

**Table 2:** Pathological processes and disease states associated with dysregulated miRNAs after ICH.

MicroRNA	Cellular/Pathological Process	Disease State
miR-122-5p	Cell growth and proliferation, inflammation, oxidative stress, apoptosis	Gastric cancer [38], renal cell carcinoma [39], liver cancer [40], pancreatic ductal adenocarcinoma [41], transient ischemic attack [42]
miR-122-3p	Cell proliferation, apoptosis, cellular stress response	Chronic atrophic gastritis [43], hepatotoxicity [44]
miR-9-3p	Apoptosis, cell proliferation, oxidative stress	Traumatic brain injury [45], ischemic stroke [46], hypoxia [47]
miR-9-5p	Apoptosis, inflammation, cell growth and proliferation, autophagy	Traumatic brain injury [45], ischemic stroke [46,48], Alzheimer’s disease [49], glioblastoma [50]
miR-137-3p	Oxidative stress, neuron necrosis, apoptosis	Lung cancer [51], ICH [52], traumatic brain injury [45], brachial plexus root avulsion [53]
miR-1298-5p	Proliferation, cell migration and adhesion, apoptosis, neuroinflammation	Breast cancer [54], non-small cell lung cancer [55], ischemic stroke[56], glioma [57]
miR-219a-2-3p	Oxidative stress, cell growth, apoptosis, neuroinflammation	Traumatic brain injury [58], lung cancer [59], spinal cord injury [60]
miR-384-5p	Autophagy, inflammation	Neurotoxicity [61], lung injury [62], spinal cord injury [63], diabetic encephalopathy [64]
miR-34b-3p	Metastasis, oxidative stress, cell growth	Concussion [65], leptomeningeal metastasis [66], colorectal cancer [67], renal cell carcinoma [68], breast invasive ductal carcinoma [69]
miR-124-3p	Inflammation, neuronal autophagy, apoptosis	Traumatic brain injury [70,71], ischemic stroke [72], gastric cancer [73], ICH [74,75,76,77]
miR-34c-5p	Neuroinflammation, growth, metabolism	Drug-resistant epilepsy [78], COPD [79], papillary thyroid carcinoma [80]
miR-34b-5p	Oxidative stress, apoptosis, inflammation, migration, proliferation, invasion	Parkinson’s disease [81], white matter ischemic injury [82], kidney injury [83], retinoblastoma [84], B- cell acute lymphoblastic leukemia [85]
miR-200b-3p	Cell growth, proliferation, metastasis	Prion disease [86], transient ischemic attack [87], colorectal cancer [88], traumatic brain injury [89]
miR-135a-5p	Apoptosis, inflammation, metastasis, proliferation, migration	Atherosclerosis [90], temporal lobe epilepsy [91], ischemic brain injury [92], colorectal cancer [93,94], diabetic nephropathy [95]
miR-148a-5p	Inflammation, cell growth, proliferation, metabolism	Irritable bowel syndrome [96], Crohn’s disease [97]
miR-133b-3p	Inflammation, neurodegeneration	Parkinson’s disease [98], Central post stroke pain[99], myotonic dystrophy [100]
miR-1199-5p	Migration, metastasis	Tumor metastasis [101]
miR-133a-5p	Apoptosis	Hepatic ischemia [102]
miR-451a	Blood brain barrier permeability, cell differentiation, metastasis, inflammation, apoptosis	Cerebral ischemia [103], ICH [104], blood brain barrier dysfunction [105], glioblastoma [106], prostate cancer [107], pancreatic cancer [108], Alzheimer’s disease [109], multiple sclerosis [110]
miR-138-5p	Neuroinflammation, metastasis	Breast cancer [111], cognitive impairment [112], glioblastoma [113], Parkinson’s disease [114]
let-7g-5p	Cell growth and differentiation, proliferation, metastasis	Alzheimer’s disease [115], gastric cancer [116], liver disease [117], glioblastoma [118]
miR-301a-3p	Inflammation, apoptosis, cell differentiation	Multiple sclerosis [119], endometriosis [120], macular degeneration [121], esophageal squamous cell carcinoma [122], heart failure [123]
miR-200c-5p	Proliferation, migration	Human hepatocellular carcinoma [124], renal cell carcinoma [125]
miR-770-3p	Metabolism	Aging [126]
miR-541-5p	Apoptosis	Carcinogenesis [127]
miR-194-5p	Proliferation, apoptosis	Epilepsy [128,129], pancreatic cancer [130], breast cancer [131], esophageal adenocarcinoma [132], mitochondrial neuro-gastrointestinal encephalomyopathy [133],
miR-200c-3p	Metastasis, inflammation, cell growth	Bladder cancer [134,135], colorectal cancer [136,137,138], ovarian cancer [139], knee osteoarthritis [140]
miR-216a-5p	Inflammation, cell growth, metastasis, autophagy	Alzheimer’s disease [141], acute pancreatitis [142], pancreas injury [143], pituitary tumors [144]

## Data Availability

The data presented in this study are available on request from the corresponding author.
